# DNA mismatch repair deficiency accelerates lung neoplasm development in *K-ras*^*LA1/+*^ mice: a brief report

**DOI:** 10.1002/cam4.420

**Published:** 2015-03-14

**Authors:** Charlene M Downey, Frank R Jirik

**Affiliations:** Department of Biochemistry and Molecular Biology, University of Calgary3280 Hospital Drive NW, Calgary, Alberta, Canada, T2N 4Z6

**Keywords:** Adenocarcinoma, DNA mismatch repair, *K-ras*, *Msh2*, NSCLC

## Abstract

Inherited as well as acquired deficiencies in specific DNA mismatch repair (MMR) components are associated with the development of a wide range of benign and malignant neoplasms. Loss of key members such as MSH2 and MLH1 severely cripples the ability of the cell to recognize and correct such lesions as base:base mismatches and replicative DNA polymerase errors such as slippages at repetitive sequences. Genomic instability resulting from MMR deficiency not only predisposes cells to malignant transformation but may also promote tumor progression. To test the latter, we interbred *Msh2*^*−/−*^ mice with the *K-ras*^*LA1/+*^ transgenic line that spontaneously develops a range of premalignant and malignant lung lesions. Compared to *K-ras*^*LA1/+*^mice, *K-ras*^*LA1/+*^; *Msh2*^*−/−*^ mice developed lung adenomas and adenocarcinomas at an increased frequency and also demonstrated evidence of accelerated adenocarcinoma growth. Since MMR defects have been identified in some human lung cancers, the mutant mice may not only be of preclinical utility but they will also be useful in identifying gene alterations able to act in concert with Kras mutants to promote tumor progression.

## Introduction

Non-small-cell lung cancer (NSCLC) is the leading cause of cancer-related mortality in the world. One of the most common oncogenic mutations in NSCLC involves activation of the *KRAS* proto-oncogene, an event seen in 25–30% of these human cancers 1–3. Mirroring this, a *Kras*-mutant mouse (designated *K-ras*^*LA1/+*^) demonstrates stochastic acquisition of an activating *Kras* G12D mutation as a result of random DNA recombination events in vivo 2. This leads primarily to the development of spontaneous multifocal lung epithelial hyperplasias, adenomas, and adenocarcinomas that are present at various stages of development in older animals 2,4.

Loss of MSH2, a key component of the DNA mismatch repair (MMR) system results in cancer predisposition in both mice and humans due to the ensuing increase in genomic instability 5,6. For example, using a transgenic mutational reporter system, we found that *Msh2*^*−/−*^ mice demonstrated up to a 15-fold elevation in mutation frequency in specific tissues 6. Humans with germline mutations of genes encoding MMR components also show increased mutagenesis, as shown by increased microsatellite instability (MSI), as exemplified by hereditary nonpolyposis colon cancer 7. Interestingly, reduced expression of *MSH2* or *MLH1* genes has been observed in over 50% of lung adenocarcinomas, and was correlated with decreased overall survival due to increases in MSI and a generalized “mutator” phenotype 8–10. Herein, we report that MMR deficiency accelerates lung tumor development in *K-ras*^*LA1/+*^ mice.

## Materials and Methods

### Genetic crosses

Transgenic *K-ras*^*LA1/+*^ mice 1,2,11 were on an 129/Sv background (provided by G. Lozano, MD Anderson Cancer Center) 1,2,11. *Msh2*^*+/−*^ mice 7 were backcrossed onto a 129/Sv genetic background (*N* = 10), and then interbred with *K-ras*^*LA1/+*^ mice to generate *K-ras*^*LA1/+*^; *Msh2*^*−/−*^ mice. Confirmation of genotype was obtained from isolation of DNA from tail tip clippings taken immediately postweaning for both the *K-ras*^*LA1/+*^ and *Msh2*^*+/−*^ transgenes. *K-ras*^*LA1/+*^ primer sequences and polymerase chain reaction (PCR) conditions were as follows: 5LA1 (5′-GACTGCTCTCTTTCACCTCC-3′), 3LA1 (5′-TGCACAGCTTAGTGAGACCC-3′), and 3MUT (5′-GGAGCAAAGCTGCTATTGGC-3′) primers were pooled into each sample, and the reaction was run for 35 cycles at 60°C annealing temperature. Band sizes were 200 bp for the wild-type (wt) allele, and 400 bp for the mutant allele. To confirm knockout of *Msh2*, reaction conditions were as follows: U771 (5′-GCTCACTTAGACGCCATTGT-3′), L926 (5′-AAAGTGCACGTCATTTGGA-3′), and L1211 (5′-GCCTTCTTGACGAGTTCTTC-3′) primers were pooled and the PCR was run for 40 cycles at 58°C annealing temperature. Band sizes were 175 bp for the wt allele, and 460 bp for the knockout allele. Animals were housed in micro-isolator cages with a 12-h light/dark cycle in a barrier facility with animal ethics approval by University of Calgary Animal Care Committee (protocol # M10063), and following Canadian Council on Animal Care guidelines. Pico-Vac Lab Mouse Diet (#5062) and water were given ad libitum.

### Mouse necropsy, lung tumor staining, and quantification

Following CO_2_ euthanasia, lungs were inflated and fixed with 10% neutral buffered formalin (NBF) delivered via the trachea. After fixation, lungs were separated into the left, apical, azygous, diaphragmatic, and cardiac lobes. Visible tumors counted prior to paraffin embedding and sectioning. One section at a depth of 100 *μ*m was stained with hematoxylin and eosin stain (H&E). Tumors were quantified and histopathology determined 1. For immunohistochemical staining, anti-Ki-67 (1:100) antibody was used (Abcam ab15580, Toronto, ON, Canada), followed by Vectastain IgG secondary (ABC kit, Vector Laboratories, Burlington, ON, Canada), and DAB substrate development (Sigma-Aldrich, Oakville, ON, Canada). Hematoxylin served as the counterstain. Microscopic images were obtained using a Leica Biosystems ScanScope CSO (Concord, ON, Canada).

### Statistics

Samples were compared using either a two-way analysis of variance (ANOVA) with Bonferroni's posttest, or one-way ANOVA (Kruskal–Wallis nonparametric test) with Dunn's multiple comparisons posttest and a test for linear trends. All statistics were calculated using GraphPad Prism v5.0b. *P* values <0.05 were considered significant.

## Results

### Increased tumor number in *K-ras*^*LA1/+*^; *Msh2*^**−*****/*****−**^ mice

**Figure 1:**
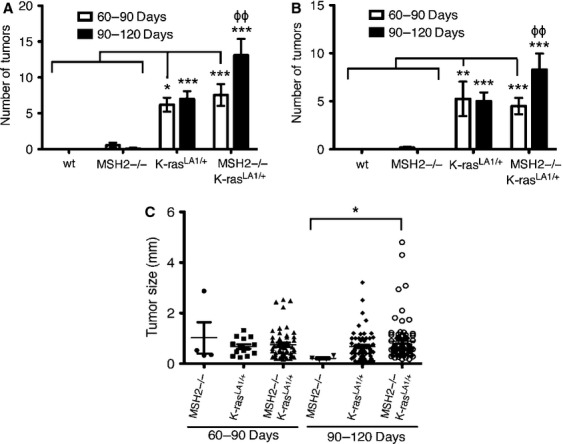
Acceleration of tumor development in *K-ras*^*LA1/+*^; *Msh2*^*−/−*^ mice. Wild-type (wt), *Msh2*^*−/−*^, *K-ras*^*LA1/+*^, and *K-ras*^*LA1/+*^; *Msh2*^*−/−*^ mice were aged to either 60–90 or 90–120 days (mean age = 73 and 99 days, respectively). (A) Macroscopically visible tumors on the lung surfaces (>1 mm diameter) were counted for all lung lobes. (B) Histology count of tumors on a single section through the center of all lung lobes. (C) Tumor diameters indicated a modest trend toward increased tumor size in the *K-ras*^*LA1/+*^; *Msh2*^*−/−*^ mice, however, this was not significant. Data are shown as the mean ± SEM, with a minimum of *N* = 10 mice per experimental group with the exception of wt (*N* = 4). Statistical analyses employed either a two-way analysis of variance (ANOVA) with Bonferroni posttest (A and B), or a one-way ANOVA (nonparametric) with tests for linear trend (C) (*P ≤* *0.05, **P ≤* *0.01, ***P ≤* *0.001 for comparisons between genotypes; ϕϕ*P *≤* *0.01 for comparisons between time points).

**Figure 2:**
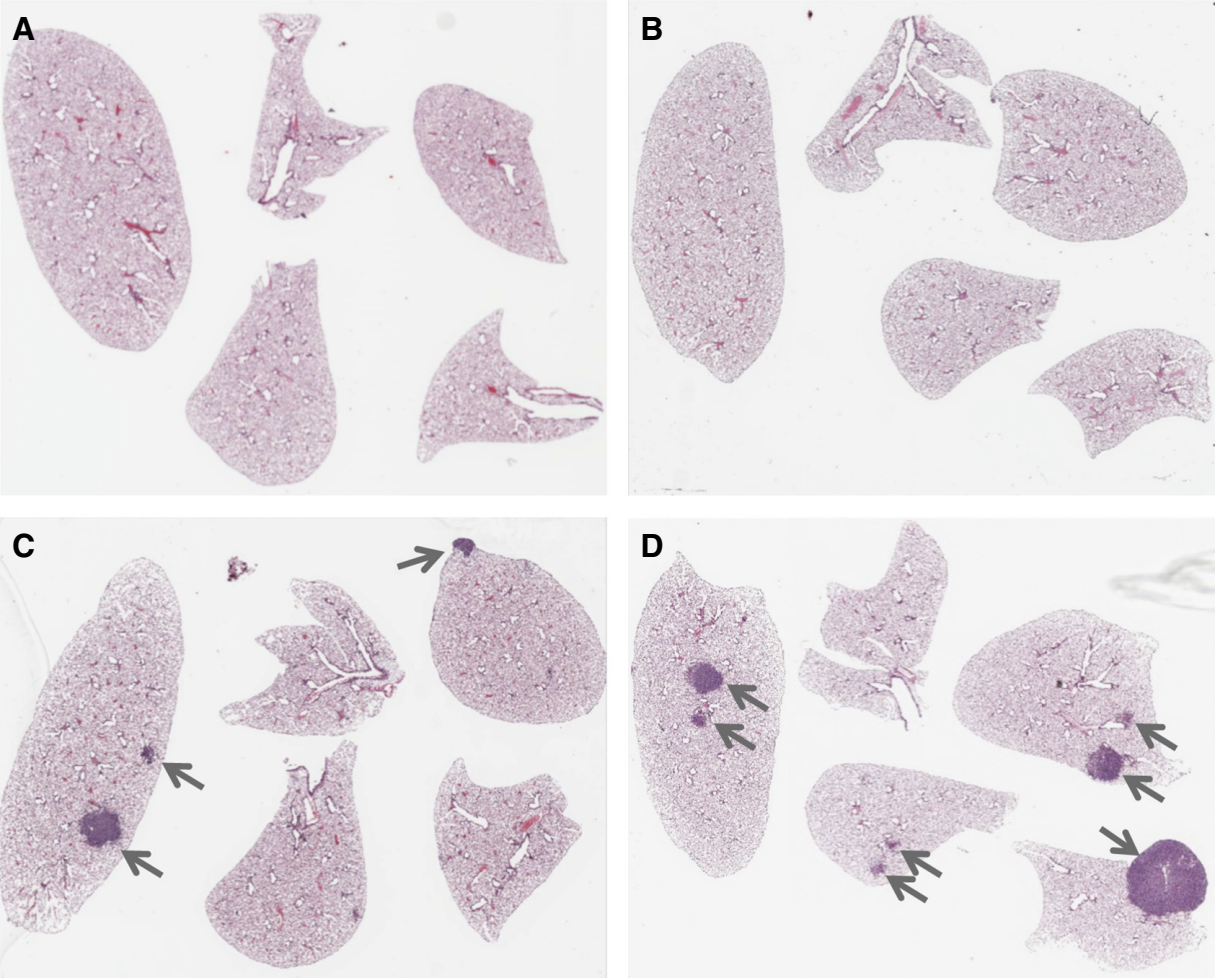
Whole lung pathology of *K-ras*^*LA1/+*^ *Msh2*^*−/−*^ mice. Whole slide images of wild-type (wt) (A), *Msh2*^*−/−*^ (B), *K-ras*^*LA1/+*^ (C), and *K-ras*^*LA1/+*^; *Msh2*^*−/−*^ (D) lungs (clockwise, beginning from the left: left, cardiac, apical, azygous, and diaphragm lobes), demonstrating the increased frequency and size of tumors in *K-ras*^*LA1/+*^; *Msh2*^*−/−*^ mice.

A count of grossly visible (macroscopic) tumors on the surfaces of the lungs (>1 mm in diameter) indicated that both *K-ras*^*LA1/+*^ and *K-ras*^*LA1/+*^; *Msh2*^*−/−*^ mice had significantly increased tumor burden compared to wt or *Msh2*^*−/−*^ genotypes alone at both 60–90 and 90–120 days old time points, while *Msh2*^*−/−*^ mice exhibited a very low frequency of lung tumor development at both time points (Fig. 1A). However, the double mutant *K-ras*^*LA1/+*^; *Msh2*^*−/−*^ mice had a significantly greater tumor burden than *K-ras*^*LA1/+*^ mice at 90–120 days (mean age = 99 days). In addition, the *K-ras*^*LA1/+*^; *Msh2*^*−/−*^ mice were the only group that demonstrated significant increases in tumor burden between 60–90 and 90–120 days old time points surveyed within the same genotype (Fig. 1A). A representative slide from the center of the lung lobes from each mouse was used to assess microscopic tumor numbers (Figs.1B and 2). Again, a significant increase in tumor formation was present in the 90–120 days old *K-ras*^*LA1/+*^; *Msh2*^*−/−*^ mice as compared to all other groups, including the 60–90 days old *K-ras*^*LA1/+*^; *Msh2*^*−/−*^ mice.

### Accelerated NSCLC development in *K-ras*^*LA1/+*^; *Msh2*^**−*****/*****−**^ mice

**Figure 3:**
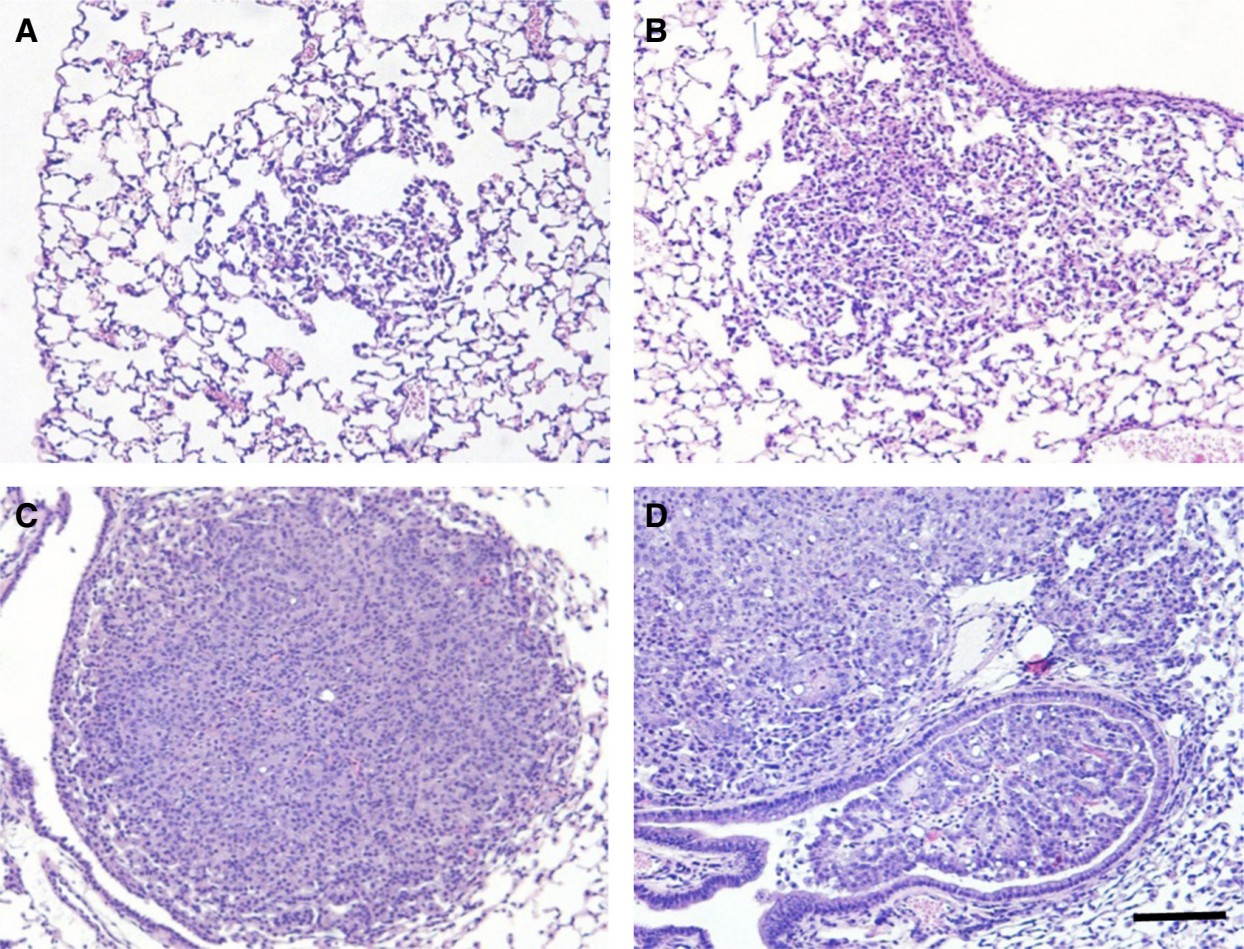
Tumor progression in *K-ras*^*LA1/+*^ *Msh2*^*−/−*^ mice. Representative hematoxylin and eosin stain (H&E) histopathology showing that tumor progression within aging *K-ras*^*LA1/+*^; *Msh2*^*−/−*^ mice follows the same progression as typically seen in *K-ras*^*LA1/+*^ mice, with localized hyperplasia (A), progressing to atypical adenomatous hyperplasia (AAH) (B), followed by adenoma (C), and ultimately adenocarcinoma (D). Unlike *K-ras*^*LA1/+*^ mice, adenocarcinomas in the 90–120 days *K-ras*^*LA1/+*^; *Msh2*^*−/−*^ group exhibited bronchoinvasion (D), perhaps consistent with accelerated progression. Scale bar = 100 *μ*m.

**Figure 4:**
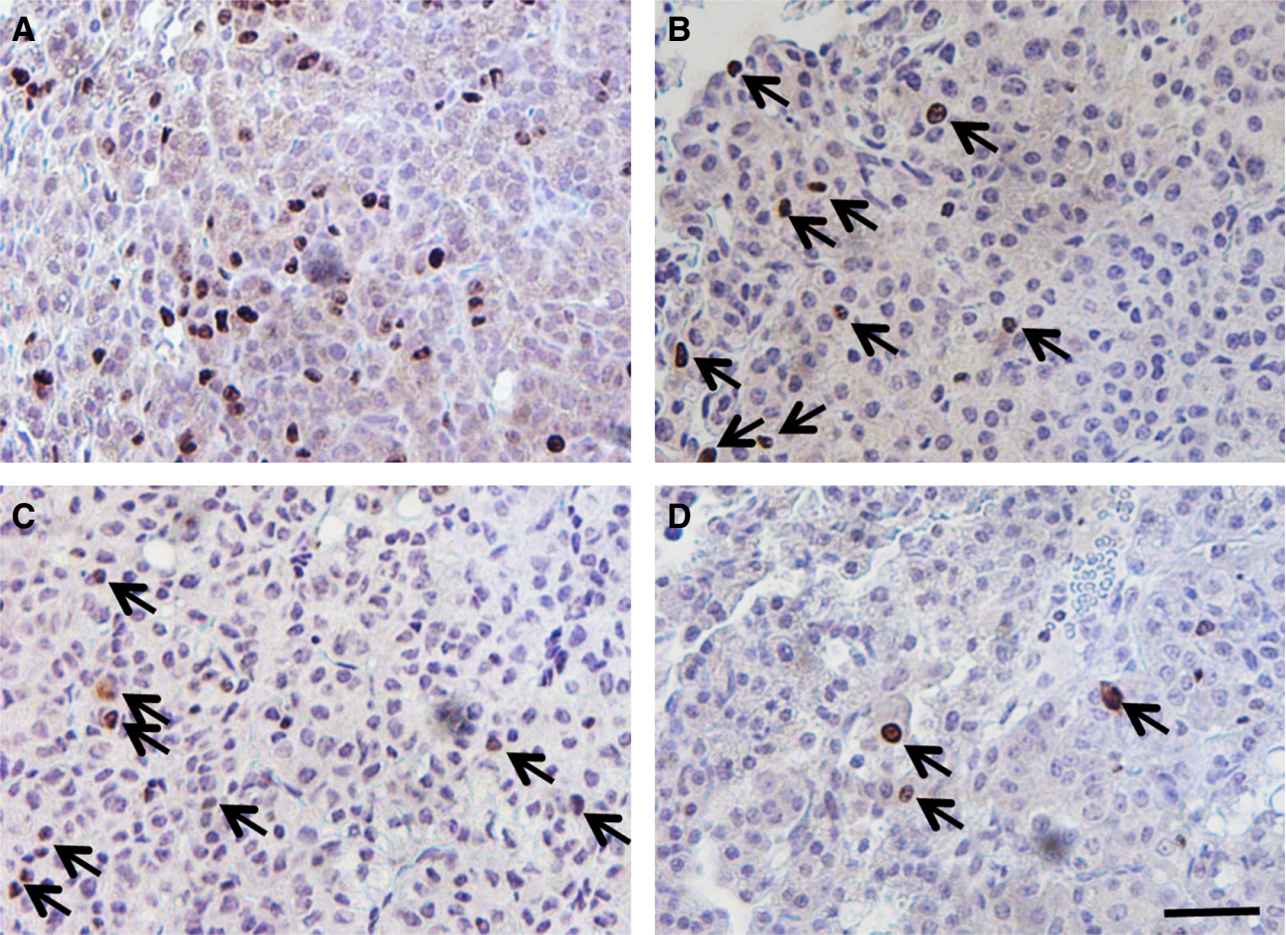
Increased tumor cell proliferation in *K-ras*^*LA1/+*^ *Msh2*^*−/−*^ mice. Representative slides were stained with an antibody to Ki67 (brown stain and arrowheads; blue hematoxylin counterstain). (A) *K-ras*^*LA1/+*^; *Msh2*^*−/−*^ 90–120 days old mice showed a clear increase in tumor cell proliferation compared with all other groups including the younger *K-ras*^*LA1/+*^; *Msh2*^*−/−*^ 60–90 days old mice (B). (C) Tumors within *K-ras*^*LA1/+*^ 90–120 days old mice and (D) *K-ras*^*LA1/+*^ 60–90 days old mice contained much lower levels of Ki67-positive cells. Scale bar = 25 *μ*m.

Tumor diameters, measured as an indicator of tumor growth and development, revealed that 90–120 days old *K-ras*^*LA1/+*^; *Msh2*^*−/−*^ mice showed a trend toward increased tumor size (although no statistical linear trend was observed), compared with *K-ras*^*LA1/+*^ mice, indicative of accelerated NSCLC growth (Fig. 1C). *K-ras*^*LA1/+*^; *Msh2*^*−/−*^ mice showed similar pathology to *K-ras*^*LA1/+*^ mice 1, from mild hyperplasia, progressing to adenoma and adenocarcinoma, with the exception of invasive adenocarcinomas present at 100 days in the *K-ras*^*LA1/+*^; *Msh2*^*−/−*^ mice (Fig. 3), a feature not commonly seen in *K-ras*^*LA1/+*^ until the latter were closer to 180 days of age (data not shown). Tumor quantification of *K-ras*^*LA1/+*^; *Msh2*^*−/−*^ mice demonstrated a significant acceleration in lung tumor development (Table1), with a higher incidence of adenocarcinomas and a statistically significant increase in adenoma formation as compared to all other groups of mice. There was an increase in Ki67-positive cells in tumors of 90–120 days old *K-ras*^*LA1/+*^; *Msh2*^*−/−*^ mice, as compared with tumors in mice of the same genotype when these were analyzed at an earlier age (60–90 days) (Fig. 4).

**Table 1:** Average occurrence of lung lesions and their respective histopathology in *K-ras*^*LA1/+*^ and *Msh2*^*−/−*^; *K-ras*^*LA1/+*^ mice at different time points

Genotype	Age	Hyperplasia	AAH	Adenoma	AdenoCA
*K-ras*^*LA1/+*^	60–90 days	0.8 (0.49)	0.6 (0.4)	2.6 (0.98)	0.2 (0.2)
	90–120 days	0.56 (0.27)	1.38 (0.36)	2.81 (0.62)	0.25 (0.19)
*Msh2*^*−/−*^; *K-ras*^*LA1/+*^	60–90 days	0.23 (0.12)	0.92 (0.33)	2.61 (0.57)	0.38 (0.18)
	90–120 days	0.44 (0.34)	1.11 (0.35)	6.33 (1.27)*	0.89 (031)

Data represented as average number of tumor subtype per mouse lung with standard error of the mean (SEM) in parentheses. AAH, atypical adenomatous hyperplasia; AdenoCA, adenocarcinoma. Minimum *N* = 10 per group.

* *P* ≤ 0.001; two-way ANOVA with Bonferroni posttest.

## Discussion

Although *K-ras*^*LA1/+*^ mice rapidly develop a range of lung tumors, we found an acceleration of lung adenocarcinoma development in *K-ras*^*LA1/+*^; *Msh2*^*−/−*^ mice by ∽120 days of age. Comparable to our results, deficiencies of two DNA repair genes (*Myh*^*−/−*^ and *Ogg1*^*−/−*^) predisposed mice to lung tumors, and this was attributed largely to acquired *K-ras*^*G12D*^ mutations 12.

The complex nature of the genomic instability that characterizes *Msh2-*null mice might suggest that in the double mutant *K-ras*^*LA1/+*^; *Msh2*^*−/−*^ mice, either *K-ras*^*LA1/+*^ recombination events were occurring at a higher frequency than in mice with *K-ras*^*LA1/+*^ alone, or that additional mutations in key growth control genes were stimulating lung tumor development and possibly progression. The cause of the tumor acceleration was likely due to acquisition of additional pro-oncogenic mutations within the tumor. While we cannot confirm this possibility, we observed an increase in Ki67-positive cells in the 90–120 days old *K-ras*^*LA1/+*^; *Msh2*^*−/−*^ tumors, perhaps indicative of a cell cycle checkpoint defect. For example, it is plausible that *Msh2*^*−/−*^ deficiency in the *K-ras*^*LA1/+*^ mice accelerated the acquisition of mutations within the *p53* gene, or other genes such as the TGF-*β* receptor 6, that could potentially play a role in promoting adenocarcinoma development.

Herein, we present evidence that DNA MMR deficiency can act in concert with one of the most frequently activated oncogenes in NSCLC, *K-ras*, to enhance lung tumor development. In humans, reports of reduced *hMLH1* and *hMSH2* expression in NSCLC have varied from ∽18% to 61% and these decreases have typically been attributed to epigenetic silencing. Furthermore, losses of *hMSH2* (and other MMR proteins, such as *hMLH1*), have correlated with an overall poor prognosis 13,14. This mouse model may provide the impetus required for addressing lung cancer formation in this population, in addition to providing a means for studying the tumor progression and targeted therapeutics.
